# Comparison of Long‐Term Health‐Related Quality of Life After Mastectomy and Breast Conserving Therapy

**DOI:** 10.1002/wjs.70517

**Published:** 2026-09-02

**Authors:** Sanna Järvinen, Tiina Jahkola, Hanna Ihalainen, Jussi P. Repo, Susanna Kauhanen, Pauliina Homsy

**Affiliations:** ^1^ Division of Musculoskeletal and Plastic Surgery Department of Plastic Surgery University of Helsinki and Helsinki University Hospital Helsinki Finland; ^2^ Division of Breast Surgery Comprehensive Cancer Center Helsinki University Hospital and University of Helsinki Helsinki Finland; ^3^ Unit of Musculoskeletal Disease Department of Orthopedics and Traumatology Tampere University Hospital and University of Tampere Tampere Finland; ^4^ Department of Orthopaedics and Traumatology Unit of Surgery Wellbeing Services County of Kanta‐Häme Hämeenlinna Finland

## Abstract

**Background:**

Breast cancer and its treatments have a significant impact on patients' health‐related quality of life (HRQL). The aim of this study was to compare HRQL in a long‐term, cross‐sectional assessment after breast conserving therapy (BCT) and mastectomy without reconstruction, using the BREAST‐Q.

**Materials and Methods:**

Altogether 500 patients who had undergone either mastectomy or BCT between 06/2016 and 8/2018 were approached. The postoperative HRQL was assessed using the BREAST‐Q Breast Conserving Therapy and Mastectomy Modules.

**Results:**

A total of 171 (34%) women participated, 85 of whom had undergone BCT and 86 mastectomy without reconstruction. The mean (SD) time from surgery to the survey was 3.3 (1) years. The mean age of the participants did not differ in the groups (*p* = 0.45). Compared to those who had undergone mastectomy, women with a history of BCT reported better psychosocial well‐being (78 (19) vs. 66 (17), *p* < 0.01), sexual well‐being (61 (20) *vs*. 41 (22), *p* < 0.01) and physical well‐being of the chest (86 (16) vs. 77 (20), *p* < 0.01). They were also more satisfied with their breasts (72 (22) vs. 52 (17), *p* < 0.01) and reported fewer radiotherapy side effects (7 (1) vs. 8 (3), *p* = 0.01).

**Conclusions:**

These data suggest that the long‐term HRQL remains high after both BCT and mastectomy. However, women with a history of BCT reported higher psychosocial, sexual, and physical well‐being and greater satisfaction with their breasts than those with a history of mastectomy.

## Graphical abstract

Long‐term BREAST‐Q outcomes after breast‐conserving therapy (BCT) and mastectomy without reconstruction. Women treated with BCT reported higher psychosocial, sexual, and physical well‐being and greater satisfaction with their breasts than women treated with mastectomy.

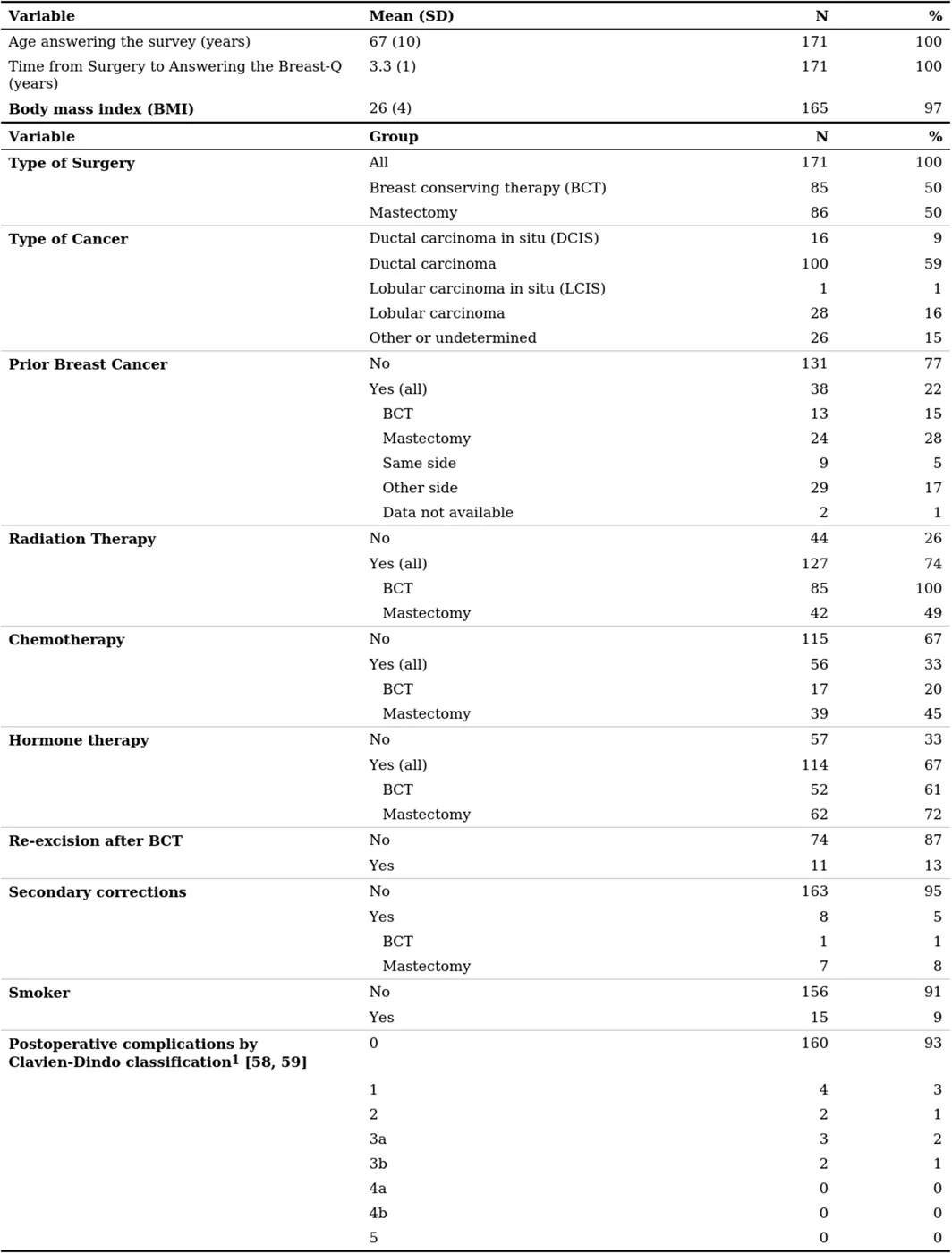

## 1 Introduction

Breast cancer is the most commonly diagnosed cancer in the world and is the leading cause of cancer mortality among females [1]. Several previous studies have demonstrated similar recurrence and survival rates between women undergoing mastectomy and BCT with radiotherapy in early‐stage breast cancer [2, 3, 4, 5]. In recent years, more attention has focused on the impact of breast cancer and its treatment on HRQL [6].

Mastectomy has been linked to lower breast satisfaction and poorer body image than BCT in previous studies [6, 7, 8, 9, 10]. Psychosocial and sexual well‐being have generally been reported to be better after BCT, although these findings are more heterogeneous [6, 7, 8, 9] and long‐term results remain limited [9, 11, 12]. Physical well‐being has shown little difference between these two surgical methods [6, 8, 9, 10] and in some studies women treated with BCT have even reported worse outcomes [7, 13]. Furthermore, in many earlier studies BCT outcomes have been compared with those of patients who had undergone mastectomy with reconstruction, or reconstructed patients have not been separated from those treated with mastectomy alone [14, 15, 16]. In the present study, we therefore focused on BCT and mastectomy without reconstruction to avoid the additional clinical heterogeneity introduced by reconstructive procedures and to isolate the impact of the two primary oncologic surgical strategies on HRQL. In addition, most of the previous studies have relied on general quality‐of‐life instruments or breast cancer–specific measures (such as EORTC QLQ‐C30 and EORTC QLQ‐BR23) [6, 10] rather than patient‐reported outcome measures specifically developed for breast surgery, such as the BREAST‐Q [7, 8, 9].

The aim of this study was to assess and compare the long‐term HRQL after BCT and mastectomy without reconstruction using the BREAST‐Q survey.

## 2 Materials and Methods

A cross‐sectional study was performed on patients who had undergone mastectomy or BCT at Helsinki University Hospital, Division of Breast Surgery, between 06/2016 and 08/2018. All women were identified from operating theater logs. Patients who had undergone immediate or delayed postmastectomy reconstruction during this period were identified but were not included in the present analysis. From the remaining cohort, a random sample of 500 women was selected and sent a postal questionnaire package, including study information, a background health form and the BREAST‐Q survey; all responses were returned in paper format. Clinical characteristics and treatment details (age, BMI, smoking status, prior breast cancer, cancer type, adjuvant and surgical treatments) were obtained from medical records.

### 2.1 Study Questionnaire

The postoperative HRQL was assessed using the Finnish Version of the BREAST‐Q (version 2.0) Breast Conserving Therapy and Mastectomy Modules. The postoperative scales used were: Psychosocial Well‐being, Sexual Well‐being, Satisfaction with Breasts, Physical Well‐being: Chest and Adverse Effects of Radiation [17, 18, 19, 20].

### 2.2 Statistical Analysis

The total score of BREAST‐Q was rescaled using a non‐linear Rasch transformation method, as per developer instructions [21]. In this method, the raw summed BREAST‐Q score was converted into a score from 0 to 100, with higher scores reflecting a better outcome. The scale of Adverse Effects of Radiation was analyzed using raw scores. The minimum score for this scale was 6 points and the maximum 18 points, with a lower score reflecting better outcomes. Missing items were handled by prorating according to the BREAST‐Q scoring manual. The answers were excluded from further analysis, if more than 50% of the answers in a subscale were missing. If less than 50% of the answers were missing, the mean response value from the other questions was used.

Analyses were performed using IBM SPSS version 29 [22]. The results presented are given as the mean value and standard deviation (SD). *p* values < 0.05 were chosen as the statistically significant level. Group comparisons used the Kruskal–Wallis test with tie correction. Correlation analysis was performed between BREAST‐Q scores and baseline patient characteristics. The age at answering the survey and BMI were examined with the Spearman's correlation coefficient. Correlations were interpreted as follows: less than 0.3: negligible; 0.3–0.5: weak; 0.5–0.7: moderate; and over 0.7: high [19, 23].

## 3 Results

Of the 500 women approached, 171 (34%) participated: 85 (50%) had undergone BCT and 86 (50%) mastectomy. Of the women with breast conserving therapy, three (4%) had undergone oncoplastic resection and the rest wide local excision. All women who had undergone BCT and 42 (49%) of those who had undergone mastectomy had received radiotherapy. No symmetrizing procedures to the contralateral side had been performed on patients who had undergone BCT. The mean (SD) time from surgery was 3.3 (1), with all patients having at least 3 years of follow‐up. The patient characteristics and treatment modalities are presented in Table 1.

**TABLE 1:** General characteristics of the study cohort.

Variable	Mean (SD)	*N*	%
Age answering the survey (years)	67 (10)	171	100
Time from surgery to answering the BREAST‐Q (years)	3.3 (1)	171	100
Body mass index (BMI)	26 (4)	165	97

Variable	Group	N	%
Type of surgery	All	171	100
	Breast conserving therapy (BCT)	85	50
	Mastectomy	86	50
Type of cancer	Ductal carcinoma in situ (DCIS)	16	9
	Ductal carcinoma	100	59
	Lobular carcinoma in situ (LCIS)	1	1
	Lobular carcinoma	28	16
	Other or undetermined	26	15
Prior breast cancer	No	131	77
	Yes	38	22
	Same side	9	5
	Other side	29	17
	Data not available	2	1
	BCT (yes)	13	15
	Mastectomy (yes)	25	29
Radiation therapy	No	44	26
	Yes	127	74
	BCT	85	100
	Mastectomy	42	49
Chemotherapy	No	115	67
	Yes	56	33
	BCT	17	20
	Mastectomy	39	45
Hormone therapy	No	57	33
	Yes	114	67
	BCT	52	61
	Mastectomy	62	72
Re‐excision after BCT	No	74	87
	Yes	11	13
Secondary corrections	No	163	95
	Yes	8	5
	BCT	1	1
	Mastectomy	7	8
Smoker	No	156	91
	Yes	15	9
Postoperative complications by clavien‐dindo classification^a^ [24, 25]	0	160	93
	1	4	3
	2	2	1
	3a	3	2
	3b	2	1
	4a	0	0
	4b	0	0
	5	0	0

*Note:* Values are presented as mean (SD) or *N* (%). *N* varies due to missing data; subgroup percentages are calculated within surgical group. *p* < 0.05 was considered statistically significant.

^a^ Complications: 1: Bleeding *n* = 2, prolonged wound care (marginal necrosis) *n* = 2; 2: Infection (treated with antibiotics) *n* = 2; 3a: Intervention not under general anesthesia: revision *n* = 3; 3b: Intervention under general anesthesia: revision (infection) *n* = 1, hematoma evacuation *n* = 1.

### 3.1 Psychosocial Well‐Being

The mean (SD) score for Psychosocial Well‐being scale for all women in our study population was 72 (19). Women with a history of BCT reported higher psychosocial well‐being compared with those who had undergone mastectomy (78 (19) vs. 66 (17), *p* < 0.01; Table 2).

**TABLE 2:** BREAST‐Q scores across different HRQL domains, comparing outcomes between breast conserving therapy and mastectomy.

	Type of surgery							
	All		BCT		Mastectomy		Group comparison	Answer rate
	*N*	Mean (SD)	*N*	Mean (SD)	*N*	Mean (SD)	*p*	%
Psychosocial well‐being	170	72 (19)	84	78 (19)	86	66 (17)	< 0.01	99
Sexual well‐being	146	51 (23)	71	61 (20)	75	41 (22)	< 0.01	85
Satisfaction with breasts	164	61 (22)	78	72 (22)	86	52 (17)	< 0.01	96
Physical well‐being: Chest	167	82 (18)	83	86 (16)	84	77 (20)	< 0.01	98
Adverse effects of radiation	111	7 (2)	80	7 (1)	31	8 (3)	0.01	65

*Note:* Values are presented as *N*, mean (SD) and *p* < 0.05 was considered statistically significant.

The history of radiotherapy did not appear to have an effect on psychosocial well‐being in patients who had undergone mastectomy (Table 3), while all BCT patients had received radiotherapy. Chemotherapy was associated with lower Psychosocial Well‐being scores when all participants were considered together (mean (SD) 68 (16) vs. 74 (20), *p* < 0.05). The effect did not hold in subgroup analyses (Tables 3 and 4). Hormone therapy had no significant effect on psychosocial well‐being in any analyses group (mean (SD) 76 (17) for patients who had received hormone therapy versus 70 (23) for those who did not receive hormone therapy; *p* = 0.08; Tables 3 and 4). Prior breast cancer had no significant effect on the Psychosocial Well‐being scores (mean (SD) 73 (18) for patients without prior breast cancer vs. 67 (19) for those with prior breast cancer; *p* = 0.11; Tables 3 and 4).

**TABLE 3:** Impact of clinical and treatment‐related factors on BREAST‐Q scores among women with history of mastectomy.

	Age answering the survey		BMI		Prior breast cancer		Radiation therapy				Chemotherapy		Hormone therapy			
	Mean (SD) 68 (11)		Mean (SD) 26 (4)		Yes (*N* = 24)	No (*N* = 58)	*p*	Yes (*N* = 41)	No (*N* = 43)	*p*	Yes (*N* = 37)	No (*N* = 47)		Yes (*N* = 61)	No (*N* = 23)	*p*
	ρ	*p*	*ρ*	*p*	Mean			Mean			Mean		*p*	Mean		
Psychosocial well‐being	0.06	0.57	−0.20	0.07	63	67	0.33	65	62	0.55	65	67	0.53	66	67	0.73
Sexual well‐being	−0.13	0.28	−0.11	0.34	38	43	0.31	41	42	0.91	43	40	0.67	40	46	0.33
Satisfaction with breasts	−0.06	0.58	−0.24	0.03	48	54	0.11	50	55	0.50	49	55	0.36	51	55	0.88
Physical well‐being: Chest	0.10	0.36	−0.12	0.29	80	76	0.46	71	83	0.03	84	69	< 0.01	75	77	0.88
Adverse effects of radiation	−0.21	0.25	0.16	0.39	8	8	0.49									

*Note:* Values are presented as *N* (*N* varies due to missing BREAST‐Q responses; group sizes shown are complete cases for each analysis), mean (without SD for readability) and Spearman's *ρ* unless otherwise stated. *p* < 0.05 was considered statistically significant.

**TABLE 4:** Impact of clinical and treatment‐related factors on BREAST‐Q scores among women with history of BCT.

	Age answering the survey		BMI		Prior breast cancer			Chemotherapy			Hormone therapy		
	Mean (SD) 67 (10)		Mean (SD) 26 (4)		Yes (*N* = 12)	No (*N* = 71)	*p*	Yes (*N* = 17)	No (*N* = 66)	*p*	Yes (*N* = 52)	No (*N* = 33)	*p*
	*ρ*	*p*	*ρ*	*p*	Mean			Mean			Mean		
Psychosocial well‐being	0.08	0.48	−0.35	< 0.01	75	79	0.94	74	79	0.20	75	82	0.11
Sexual well‐being	0.06	0.65	−0.22	0.08	65	60	0.56	55	63	0.13	60	64	0.30
Satisfaction with breasts	0.18	0.12	−0.35	< 0.01	78	76	0.93	67	73	0.30	70	74	0.43
Physical well‐being: Chest	0.25	0.03	−0.002	0.99	96	84	< 0.01	87	86	0.79	86	86	0.95
Adverse effects of radiation	−0.24	0.02	0.24	0.04	7	7	0.65						

*Note:* Values are presented as *N* (*N* varies due to missing BREAST‐Q responses; group sizes shown are complete cases for each analysis), mean (without SD for readability) and Spearman's *ρ* unless otherwise stated. *p* < 0.05 was considered statistically significant.

### 3.2 Sexual Well‐Being

The mean (SD) value for Sexual Well‐being for all women was 51 (23). The Sexual Well‐Being was higher in women who had undergone BCT than in those who had undergone mastectomy (61 (20) vs. 41 (22), *p* < 0.01; Table 2).

Sexual well‐being was not affected by the history of radiotherapy in women who had undergone mastectomy (Table 3). Chemotherapy was associated with lower scores when all patients were considered together (mean (SD) 54 (24) for those who had undergone chemotherapy vs. 46 (22) for those who had not; *p* = 0.04). The effect was not observed in subgroup analyses of women with a history of BCT or mastectomy (Tables 3 and 4). Hormone therapy had no effect on Sexual Well‐being value in any analysis group (mean (SD) 49 (23) for patients receiving hormone therapy vs. 56 (23) for those not receiving hormone therapy; *p* = 0.07, Tables 3 and 4). There was no significant difference in sexual well‐being between patients with and without prior breast cancer (mean (SD) 52 (23) for those without prior breast cancer vs. 47 (23) for those with prior breast cancer, *p* = 0.14; Tables 3 and 4).

### 3.3 Satisfaction With Breasts

The mean (SD) score for all participants was 61 (22). Women with a history of BCT reported higher satisfaction with the breasts than those who had undergone mastectomy (72 (22) vs. 52 (17), *p* < 0.01; Table 2).

Radiotherapy had no significant effect on the Satisfaction with Breasts scores in mastectomy group (Table 3). Hormone therapy, chemotherapy and prior breast cancer had no effect on satisfaction with breasts scores in any group analysis (Tables 3 and 4). The questionnaire used different postoperative forms for breast satisfaction in patients who had undergone BCT and those who had undergone mastectomy, therefore only subgroup analyses were performed for this scale (Tables 3 and 4).

### 3.4 Physical Well‐Being: Chest

The mean (SD) value for all patients was 82 (18). The mean value for Physical Well‐being: chest was higher in patients who had undergone BCT compared with those who had undergone mastectomy (mean (SD) 86 (16) vs. 77 (20), *p* < 0.01; Tables 3 and 4).

The history of radiotherapy was associated with lower physical well‐being of the chest in patients with a history of mastectomy (mean (SD) 71 (23) vs. 83 (14), *p* = 0.03; Table 3), while all BCT patients had received radiotherapy.

Chemotherapy was associated with lower Physical Well‐being: Chest scores when all participants considered together (mean (SD) 74 (23) vs. 85 (15); *p* < 0.01). This result was observed within the mastectomy group but not detected in women with a history of BCT (Tables 3 and 4). Hormone therapy had no effect on Physical Well‐being: chest scale score (mean (SD) 80 (20) vs. 85 (15) for all women, *p* = 0.13; Tables 3 and 4).

Prior breast cancer had no effect on Physical Well‐being: chest scale score considered all patients together (mean (SD) 81 (19) for patients without prior breast cancer vs. 85 (16) for those with prior breast cancer, *p* = 0.17), nor for mastectomy patients considered separately (Table 3). For women with a history of BCT, prior breast cancer was associated with higher physical well‐being of the chest compared with no prior cancer (mean (SD) 98 (6) vs. 84 (16) *p* < 0.01; Table 4).

### 3.5 Adverse Effects of Radiation

Radiotherapy was given to 127 patients, all the patients who had undergone BCT (*n* = 85) and 42 (49%) patients who had undergone mastectomy. The mean (SD) score for Adverse Effects of Radiation scale for all patients was 7 (2) among irradiated patients. Reported adverse effects were low across all analysis groups. Women who had undergone BCT reported fewer adverse effects compared with those who had undergone mastectomy (mean (SD) 7 (1) vs. 8 (3), *p* = 0.01; Table 2). Prior breast cancer had no effect on the Adverse Effects of Radiation scale (mean (SD) 7 (2) vs. 7 (1), *p* = 0.73; Tables 3 and 4).

### 3.6 Associations of BMI and Age Answering the Survey With BREAST‐Q

In women with a history of BCT, BMI correlated negatively with Psychosocial Well‐being and Satisfaction with Breasts (*ρ* = −0.35, *p* < 0.01 for both; Tables 3 and 4), suggesting that as the BMI increases, the Psychosocial Well‐being and Satisfaction with Breasts decreases. These associations were not observed after mastectomy. Age at answering the survey did not correlate with any of the BREAST‐Q subscale scores (Tables 3 and 4).

## 4 Discussion

Breast cancer and its surgical treatment can significantly impact HRQL [6, 26]. This study shows that, 3 years after surgery, women who had undergone BCT reported better psychosocial, sexual and physical well‐being and higher breast satisfaction than women with a history of mastectomy. The scores were comparable to, or higher than, both normative values from healthy women [20] and preoperative values for newly diagnosed breast cancer patients before the surgery [27].

Women with a history of BCT reported markedly better psychosocial well‐being compared to those who had undergone mastectomy (mean 78 vs. 66), and the difference exceeded the minimal important difference (MID), 4, indicating clinical significance [28, 29]. The BCT mean also exceeded both the normative and preoperative reference values (71 in both) [20, 27] highlighting that their psychosocial well‐being was comparable to or better than in women without cancer and in newly diagnosed patients before surgery.

Similar result with women with a history of BCT reporting better psychosocial well‐being has been previously observed [8, 9] although not consistently [7]. Previous long‐term studies using other quality of life measures, such as EORTC QLQ‐C30/Br23 and 15D, [30, 31], have suggested similar results to ours [11, 32]. Notably, two earlier Finnish studies from our own institution, conducted in different time periods and using different HRQL instruments, reported higher global HRQL and better body image after BCT than after mastectomy [33, 34]. Furthermore, the difference in the HRQL of women with a history of mastectomy and BCT appear to remain consistent over time [9, 33].

Women who had undergone BCT reported significantly higher Sexual Well‐being scores, 61, than those who had undergone mastectomy, 41 difference exceeding the MID of 4 [28, 29]. The BCT mean also surpassed both the normative value (56 [20]) and the preoperative (58 [27]) reference value. Several studies have reported similar, long‐lasting differences, [6, 9, 32, 35], although findings are heterogeneous and some have found no significant group differences [10, 11, 36]. Chemotherapy has been found to have a negative effect on sexual well‐being [26, 37], an effect observed also in our study.

Women who had undergone BCT were markedly more satisfied with their breasts than those who had undergone mastectomy (mean 72 vs. 52). The mean score also exceeded both the normative value for healthy women (58) and the preoperative reference value (60) for this scale [20, 27]. The difference clearly surpassed the MID of 4 points [28, 29]. Better breast satisfaction in women with a history of BCT compared to those who had undergone mastectomy has been reported in a previous long‐term study and studies with shorter follow‐up [7, 8, 9, 12]. The high satisfaction with overall body image has been shown to persist in BCT patients [11, 33, 38]. In addition, a broad range of studies using different HRQL instruments has shown that women treated with BCT generally report a more favorable body image than those treated with mastectomy. Furthermore, breast satisfaction and body image appear to be the domains with the most consistent evidence supporting the superiority of BCT over mastectomy [6, 10].

The physical well‐being of the chest was higher among women with a history of BCT compared to those who had undergone mastectomy (mean 86 vs. 77), and the difference exceeded the MID of 4 points [28, 29]. The mean score in women who had undergone BCT was also higher than the preoperative reference value of 81 [27], although both surgical groups scored lower than the normative reference value, 93, for healthy women [20]. Most previous studies have reported no difference in physical well‐being between the two surgical groups [6, 8, 9, 10, 36, 39, 40], although several studies have found better outcomes after BCT [11, 33], and a few have even reported superior outcomes among women who had undergone mastectomy compared to those treated with BCT [7, 13]. The lower scores observed after mastectomy may reflect the greater extent of tissue removal and potential sensory changes following surgery [41, 42]. It has been also suggested that a breast cancer diagnosis itself may increase the experience of breast‐related symptoms [43] and that increased depression and anxiety in breast cancer patients can amplify the perception of pain even before treatment [44]. In addition to surgical and psychological factors, adjuvant treatments may also influence physical well‐being. In our study, chemotherapy was associated with lower physical well‐being scores overall, although this association was not observed within the BCT group. However, the number of patients who had received chemotherapy in this group was small, 17, limiting the power of the analysis.

Adverse Effects of Radiation were few in our study population. Women with a history of BCT reported fewer radiotherapy‐related side effects than those who had undergone mastectomy (mean score 7 vs. 8). Radiotherapy was not associated with lower BREAST‐Q scores in any domain, except for physical well‐being of the chest, in which it was associated with lower scores among women who had undergone mastectomy. This finding contrasts with previous studies that have demonstrated radiation as an independent predictor of poor patient‐reported outcomes [45, 46]. Our results likely reflect the fact that radiotherapy‐related decrements in quality of life are most pronounced in the acute phase and tend to diminish over time, and that contemporary radiotherapy techniques with more focused fields may mitigate persistent late toxicity [47, 48, 49].

Higher BMI was weakly associated with lower psychosocial well‐being and satisfaction with breasts, but not with sexual well‐being or physical well‐being, among patients who had undergone BCT. Such associations were not observed among women who had undergone mastectomy. This is consistent with earlier studies in BCT cohorts showing that higher BMI is linked to lower breast satisfaction and HRQL in general. [38, 50, 51, 52, 53]. Among women with newly diagnosed breast cancer, higher BMI was associated with poorer preoperative satisfaction with breasts and worse psychosocial, sexual, and physical well‐being [27].

Age was not found to have an impact on any of the BREAST‐Q domains. Younger age has previously been associated with higher satisfaction of breasts and better sexual well‐being [46, 54], although some studies have observed no significant relationship [55, 56]. It has been also suggested that younger women experience greater psychological burden from breast cancer and its treatments [11, 40, 45, 54], possibly impacting HRQL.

Our study was limited by its retrospective design and relatively small sample size, which restricted subgroup analyses. The response rate of 34%, although typical for postal surveys [57], and the mail‐based mode of data collection may introduce selection bias and limit the generalizability of our findings. In line with the BREAST‐Q scoring manual, missing items were handled by prorating when fewer than 50% of items in a subscale were missing, but this imputation may add some uncertainty to the estimated scores. At the time of the study, some patients were likely still receiving endocrine therapy, which may have influenced certain HRQL domains.

The strengths of our study include the fact that we specifically compared breast conserving therapy with non‐reconstructed mastectomy, avoiding the heterogeneity that arises when mastectomy cohorts include a substantial proportion of patients who have undergone reconstruction. Second, we used disease‐specific BREAST‐Q modules for both BCT and mastectomy patients within the same cohort and at a clearly defined long‐term time point, more than 3 years after surgery. Third, our cohort represents a Northern European population, which has been underrepresented in earlier research. In contrast to some previous studies, where the differences between BCT and mastectomy have been more inconsistent, we observed a consistent pattern favoring BCT, with higher psychosocial, sexual and physical well‐being and greater satisfaction with the breasts in women treated with BCT compared with those who have undergone mastectomy without reconstruction.

In conclusion, this long‐term BREAST‐Q–based comparison suggests that overall HRQL remains high after both procedures. However, BCT is associated with more favorable outcomes in psychosocial, sexual and physical well‐being, and with satisfaction with breasts, than non‐reconstructed mastectomy, supporting its use when oncologically feasible.

## Author Contributions

**Sanna Järvinen:** data curation, methodology, visualization, writing – original draft, validation, formal analysis, investigation. **Tiina Jahkola:** conceptualization, methodology, writing – review and editing. **Hanna Ihalainen:** conceptualization, methodology, writing – review and editing, investigation. **Jussi P. Repo:** conceptualization, methodology, writing – review and editing. **Susanna Kauhanen:** conceptualization, methodology, writing – review and editing. **Pauliina Homsy:** conceptualization, methodology, data curation, writing – review and editing, supervision, project administration, validation.

## Funding

This work was supported by funding from Helsinki University Hospital Musculoskeletal and Plastic Surgery Research Center.

## Ethics Statement

This study was conducted in compliance with ethical principles and institutional guidelines. The study protocol was approved by the Helsinki University Hospital Ethics Committee (HUS/1780/2017). Written informed consent was obtained from all study participants.

## Conflicts of Interest

The authors declare no conflicts of interest.

## Data Availability

The data that support the findings of this study are available from the corresponding author upon reasonable request.

## References

[1] L. Wilkinson and T. Gathani , “Understanding Breast Cancer as a Global Health Concern,” British Journal of Radiology 95, no. 1130 (2022): 20211033, 10.1259/bjr.20211033. PMC8822551 34905391

[2] B. Fisher , S. Anderson , J. Bryant , et al., “Twenty‐Year follow‐up of a Randomized Trial Comparing Total Mastectomy, Lumpectomy, and Lumpectomy plus Irradiation for the Treatment of Invasive Breast Cancer,” New England Journal of Medicine 347, no. 16 (2002): 1233–1241, 10.1056/nejmoa022152. 12393820

[3] M. Clarke , R. Collins , S. Darby , et al., “Effects of Radiotherapy and of Differences in the Extent of Surgery for Early Breast Cancer on Local Recurrence and 15‐Year Survival: An Overview of the Randomised Trials,” Lancet 366 (2005): 2087–2106, 10.1016/S0140-6736(05)67887-7. 16360786

[4] U. Veronesi , N. Cascinelli , L. Mariani , et al., “Twenty‐Year Follow‐Up of a Randomized Study Comparing Breast‐Conserving Surgery With Radical Mastectomy for Early Breast Cancer,” New England Journal of Medicine 347, no. 16 (2002): 1227–1232, 10.1056/nejmoa020989. 12393819

[5] J. A. Jacobson , D. N. Danforth , K. H. Cowan , et al., “Ten‐Year Results of a Comparison of Conservation With Mastectomy in the Treatment of Stage I and II Breast Cancer,” New England Journal of Medicine 332, no. 14 (1995): 907–911, 10.1056/nejm199504063321402. 7877647

[6] S. Zehra , F. Doyle , M. Barry , S. Walsh , and M. R. Kell , “Health‐Related Quality of Life Following Breast Reconstruction Compared to Total Mastectomy and Breast‐Conserving Surgery Among Breast Cancer Survivors: A Systematic Review and Meta‐Analysis,” Breast Cancer 27, no. 4 (2020): 534–566, 10.1007/s12282-020-01076-1. 32162181

[7] B. H. Howes , D. I. Watson , C. Xu , B. Fosh , M. Canepa , and N. R. Dean , “Quality of Life Following Total Mastectomy With and Without Reconstruction Versus Breast‐Conserving Surgery for Breast Cancer: A Case‐Controlled Cohort Study,” Journal of Plastic, Reconstructive & Aesthetic Surgery 69, no. 9 (2016): 1184–1191, 10.1016/j.bjps.2016.06.004. 27406255

[8] L. M. Vohra , S. M. Javed , D. Jabeen , S. S. Abidi , and M. U. Tahseen , “Quality of Life of Breast Cancer Survivors: A Comparison of Breast Conserving Surgery Versus Total Mastectomy With and Without Immediate Reconstruction: A Prospective Cohort Study,” Annals of Medicine and Surgery 85, no. 5 (2023): 1513–1517, 10.1097/ms9.0000000000000607. PMC10205257 37228946

[9] S. Tomita , T. Yoshitake , N. Matsunaga , M. de Kerckhove , M. Fujii , and Y. Terao , “Patient‐Reported Outcomes and Quality of Life After Breast‐Conserving Surgery, Mastectomy, and Breast Reconstruction Assessed Using the BREAST‐Q Questionnaire,” Breast Cancer Research and Treatment 207, no. 3 (2024): 641–648, 10.1007/s10549-024-07396-6. 38874689

[10] E. T. Ng , R. Z. Ang , B. X. Tran , et al., “Comparing Quality of Life in Breast Cancer Patients Who Underwent Mastectomy Versus Breast‐Conserving Surgery: A Meta‐Analysis,” International Journal of Environmental Research and Public Health 16, no. 24 (2019): 4970, 10.3390/ijerph16244970. PMC6950729 31817811

[11] Y. Sun , S. W. Kim , C. Y. Heo , et al., “Comparison of Quality of Life Based on Surgical Technique in Patients With Breast Cancer,” Japanese Journal of Clinical Oncology 44, no. 1 (2014): 22–27, 10.1093/jjco/hyt176. 24277749

[12] P. Yu , H. Tang , Y. Zou , et al., “Breast‐Conserving Therapy Versus Mastectomy in Young Breast Cancer Patients Concerning Molecular Subtypes: A SEER Population‐Based Study,” Cancer Control 27, no. 1 (2020): 1073274820976667, 10.1177/1073274820976667. PMC8480363 33356518

[13] A. O. Cortés‐Flores , G. Morgan‐Villela , C. J. Zuloaga‐Fernández del Valle , et al., “Quality of Life Among Women Treated for Breast Cancer: A Survey of Three Procedures in Mexico,” Aesthetic Plastic Surgery 38 (2014): 887–895, 10.1007/s00266-014-0384-5. 25099496

[14] S. E. Hanson , X. Lei , M. S. Roubaud , et al., “Long‐Term Quality of Life in Patients With Breast Cancer After Breast Conservation vs Mastectomy and Reconstruction,” JAMA Surgery 157, no. 6 (2022): e220631, 10.1001/jamasurg.2022.0631. PMC9008558 35416926

[15] K. Diao , X. Lei , W. He , et al., “Patient‐Reported Quality of Life After Breast‐Conserving Surgery With Radiotherapy Versus Mastectomy and Reconstruction,” Annals of Surgery 278, no. 5 (2023): e1096–e1102, 10.1097/sla.0000000000005920. PMC10592600 37232937

[16] M. R. Flanagan , E. C. Zabor , A. Romanoff , et al., “A Comparison of Patient‐Reported Outcomes After Breast‐Conserving Surgery and Mastectomy With Implant Breast Reconstruction,” Annals of Surgical Oncology 26, no. 10 (2019): 3133–3140, 10.1245/s10434-019-07548-9. PMC6752888 31342397

[17] A. L. Pusic , A. F. Klassen , A. M. Scott , J. A. Klok , P. G. Cordeiro , and S. J. Cano , “Development of a New Patient‐Reported Outcome Measure for Breast Surgery: The BREAST‐Q,” Plastic and Reconstructive Surgery 124, no. 2 (2009): 345–353, 10.1097/prs.0b013e3181aee807. 19644246

[18] BREAST‐Q , BREAST‐Q | Breast Cancer—Q‐Portfolio Adaptable to Your Needs.

[19] P. Homsy , J. Repo , C. Kuhlefelt , et al., “Finnish Translation, Validation, and Reproducibility of BREAST‐Q Modules Relevant to Breast Cancer Treatment,” Scandinavian Journal of Surgery (2024): 14574969241277636. 10.1177/14574969241277636 39308151

[20] L. R. Mundy , K. Homa , A. F. Klassen , A. L. Pusic , and C. L. Kerrigan , “Breast Cancer and Reconstruction: Normative Data for Interpreting the BREAST‐Q,” Plastic and Reconstructive Surgery 139, no. 5 (2017): 1046e–1055e, 10.1097/prs.0000000000003241. PMC5713639 28445351

[21] S. Cano , A. Klassen , and A. Pusic , “From Breast‐Q © to Q‐Score ©: Using Rasch Measurement to Better Capture Breast Surgery Outcomes,” Acta Imeko (2011).

[22] IBM Corp , IBM SPSS Statistics for Windows, Version 29.0.2.0 (IBM Corp, 2023).

[23] M. M. Mukaka , “Statistics Corner: A Guide to Appropriate Use of Correlation Coefficient in Medical Research,” Malawi Medical Journal 24 (2012): 69–71, PMID: 2363878; PMCID: PMC3576830, https://pubmed.ncbi.nlm.nih.gov/23638278/?utm_source=chatgpt.com. PMC3576830 23638278

[24] D. Dindo , N. Demartines , and P. A. Clavien , “Classification of Surgical Complications: A New Proposal With Evaluation in a Cohort of 6336 Patients and Results of a Survey,” Annals of Surgery 240, no. 2 (2004): 205–213, 10.1097/01.sla.0000133083.54934.ae. PMC1360123 15273542

[25] P. A. Clavien , J. Barkun , M. L. de Oliveira , et al., “The Clavien‐Dindo Classification of Surgical Complications: Five‐Year Experience,” Annals of Surgery 250, no. 2 (2009): 187–196, 10.1097/sla.0b013e3181b13ca2. 19638912

[26] K. Shimozuma , P. A. Ganz , L. Petersen , and K. Hirji , “Quality of Life in the First Year After Breast Cancer Surgery: Rehabilitation Needs and Patterns of Recovery,” Breast Cancer Research and Treatment 56, no. 1 (1999): 45–57, 10.1023/a:1006214830854. 10517342

[27] C. Kuhlefelt , J. P. Repo , V. Rasi , et al., “Preoperative Reference Values for Breast Cancer Patients Using the BREAST‐Q,” Breast 78 (2024): 103832, 10.1016/j.breast.2024.103832. PMC11570751 39500222

[28] S. H. Voineskos , A. F. Klassen , S. J. Cano , A. L. Pusic , and C. J. Gibbons , “Giving Meaning to Differences in BREAST‐Q Scores: Minimal Important Difference for Breast Reconstruction Patients,” Plastic and Reconstructive Surgery 145, no. 1 (2020): 11e–20e, 10.1097/prs.0000000000006317. 31577663

[29] J. J. Chu , A. B. Tadros , L. Gallo , et al., “Interpreting the BREAST‐Q for Breast‐Conserving Therapy: Minimal Important Differences and Clinical Reference Values,” Annals of Surgical Oncology 30, no. 7 (2023): 4075–4084, 10.1245/s10434-023-13222-y. PMC10996067 36840864

[30] H. Sintonen , “The 15D Instrument of Health‐Related Quality of Life: Properties and Applications,” Annals of Medicine 33, no. 5 (2001): 328–336, 10.3109/07853890109002086. 11491191

[31] T. Young , H. De Haes , D. Curran , et al., Guidelines for Assessing Quality of Life in EORTC Clinical Trials (EORTC Quality of Life Group, 1999).

[32] S. M. Rosenberg , L. S. Dominici , S. Gelber , et al., “Association of Breast Cancer Surgery With Quality of Life and Psychosocial Well‐Being in Young Breast Cancer Survivors,” JAMA Surgery 155, no. 11 (2020): 1035–1042, 10.1001/jamasurg.2020.3325. PMC7495332 32936216

[33] M. Rautalin , T. Jahkola , and R. P. Roine , “Surgery And Health‐Related Quality of Life—A Prospective Follow Up Study on Breast Cancer Patients in Finland,” European Journal of Surgical Oncology 47, no. 7 (2021): 1581–1587, 10.1016/j.ejso.2021.02.006. 33593622

[34] T. Jahkola , “Self‐Perceptions of Women After Early Breast Cancer Surgery,” European Journal of Surgical Oncology 24, no. 1 (1998): 9–14, 10.1016/s0748-7983(98)80116-9. 9542507

[35] D. Doege , M. S. Y. Thong , L. Koch‐Gallenkamp , et al., “Clinical and Sociodemographic Determinants of Disease‐Specific Health‐Related Quality of Life in Long‐Term Breast Cancer Survivors,” Journal of Cancer Research and Clinical Oncology 148 (2022): 3461–3473, 10.1007/s00432-022-04204-w. PMC9587098 35879433

[36] J. Han , D. Grothuesmann , M. Neises , U. Hille , and P. Hillemanns , “Quality of Life and Satisfaction After Breast Cancer Operation,” Archives of Gynecology and Obstetrics 282, no. 1 (2010): 75–82, 10.1007/s00404-009-1302-y. 19960349

[37] P. A. Ganz , J. H. Rowland , K. Desmond , B. E. Meyerowitz , and G. E. Wyatt , “Life After Breast Cancer: Understanding Women's Health‐Related Quality of Life and Sexual Functioning,” Journal of Clinical Oncology 16, no. 2 (1998): 501–514, 10.1200/jco.1998.16.2.501. 9469334

[38] A. H. S. Olfatbakhsh , M. Tabari , E. Hashemi , F. Sari , and A. Kaviani , “Patient Satisfaction and Body Image Following Mastectomy, Breast‐Conserving Therapy, and Mastectomy With Reconstruction: A Study in Iran,” Archives of Breast Cancer (2018): 173–182, 10.32768/abc.201854173-182.

[39] K. A. Metcalfe , T. Zhong , S. A. Narod , et al., “A Prospective Study of Mastectomy Patients With and Without Delayed Breast Reconstruction: Long‐term Psychosocial Functioning in the Breast Cancer Survivorship Period,” Journal of Surgical Oncology 111, no. 3 (2015): 258–264, 10.1002/jso.23829. 25557452

[40] R. Spatuzzi , A. Vespa , P. Lorenzi , et al., “Evaluation of Social Support, Quality of Life, and Body Image in Women With Breast Cancer,” Breast Care 11, no. 1 (2016): 28–32, 10.1159/000443493. PMC4813650 27051393

[41] S. T. Lindau , E. A. Pinkerton , E. M. Abramsohn , et al., “Importance of Breast Sensation After Mastectomy: Evidence From Three Sources,” Women's Health Reports 4, no. 1 (2023): 594–602, 10.1089/whr.2023.0106. PMC10719638 38099078

[42] T. Zhu , Y. Jiang , T. Liu , et al., “The Altering in Sensory Sensitivity: A Current Issue of Female Breast Surgery,” International Journal of Medical Sciences 19, no. 5 (2022): 901–908, 10.7150/ijms.71913. PMC9149637 35693749

[43] J. Fortin , M. Leblanc , G. Elgbeili , M. J. Cordova , M.‐F. Marin , and A. Brunet , “The Mental Health Impacts of Receiving a Breast Cancer Diagnosis: A Meta‐Analysis,” British Journal of Cancer 125, no. 11 (2021): 1582–1592, 10.1038/s41416-021-01542-3. PMC8608836 34482373

[44] S. K. Galloway , M. Baker , P. Giglio , et al., “Depression and Anxiety Symptoms Relate to Distinct Components of Pain Experience Among Patients With Breast Cancer,” Pain Research and Treatment 2012 (2012): 851276, 10.1155/2012/851276. PMC3512324 23227331

[45] R. Jagsi , Y. Li , M. Morrow , et al., “Patient‐Reported Quality of Life and Satisfaction With Cosmetic Outcomes After Breast Conservation and Mastectomy With and Without Reconstruction: Results of a Survey of Breast Cancer Survivors,” Annals of Surgery 261, no. 6 (2015): 1198–1206, 10.1097/sla.0000000000000908. PMC4512928 25654742

[46] K. B. Santosa , J. Qi , H. M. Kim , J. B. Hamill , E. G. Wilkins , and A. L. Pusic , “Long‐Term Patient‐Reported Outcomes in Postmastectomy Breast Reconstruction,” JAMA Surgery 153, no. 10 (2018): 891–899, 10.1001/jamasurg.2018.1677. PMC6233781 29926096

[47] C. Xiao , A. H. Miller , J. Felger , D. Mister , T. Liu , and M. A. Torres , “A Prospective Study of Quality of Life in Breast Cancer Patients Undergoing Radiation Therapy,” Advances in Radiation Oncology 1 (2016): 10–16, 10.1016/j.adro.2016.01.003. PMC4950982 27453954

[48] J. J. Zhang , H. Shu , S. S. Hu , Y. Yu , Yi Sun , and Y. Lv , “Relationship Between Time Elapsed Since Completion of Radiotherapy and Quality of Life of Patients With Breast Cancer,” BMC Cancer 18, no. 1 (2018): 305, 10.1186/s12885-018-4207-y. PMC5859708 29554869

[49] Z. Brownlee , R. Garg , M. Listo , P. Zavitsanos , D. E. Wazer , and K. E. Huber , “Late Complications of Radiation Therapy for Breast Cancer: Evolution in Techniques and Risk over Time,” Gland Surgery 7, no. 4 (2018): 371–378, 10.21037/gs.2018.01.05. PMC6107587 30175054

[50] I. Stolpner , J. Heil , F. Riedel , et al., “Long‐Term Patient Satisfaction and Quality of Life After Breast‐Conserving Therapy: A Prospective Study Using the BREAST‐Q,” Annals of Surgical Oncology 28, no. 13 (2021): 8742–8751, 10.1245/s10434-021-10377-4. PMC8591011 34279753

[51] R. F. Kushner and G. D. Foster , “Obesity and Quality of Life,” Nutrition 16, no. 10 (2000): 947–952, 10.1016/s0899-9007(00)00404-4. 11054600

[52] M. de Zwaan , I. Petersen , M. Kaerber , et al., “Obesity and Quality of Life: A Controlled Study of Normal‐Weight and Obese Individuals,” Psychosomatics 50, no. 5 (2009): 474–482, 10.1016/s0033-3182(09)70840-0. 19855033

[53] M. Laxy , C. Teuner , R. Holle , and C. Kurz , “The Association Between BMI and Health‐Related Quality of Life in the US Population: Sex, Age and Ethnicity Matters,” International Journal of Obesity 42, no. 3 (2018): 318–326, 10.1038/ijo.2017.252. 28993709

[54] M. Ritter , B. M. Ling , I. Oberhauser , et al., “The Impact of Age on Patient‐Reported Outcomes After Oncoplastic Versus Conventional Breast Cancer Surgery,” Breast Cancer Research and Treatment 187, no. 2 (2021): 437–446, 10.1007/s10549-021-06126-6. PMC8189956 33606158

[55] C. D. Liao , J. Xia , K. Wang , K. Zhao , L. T. Glickman , and N. A. Agrawal , “A Systematic Review of the Impact of Patient Factors on BREAST‐Q Outcomes After Reduction Mammoplasty,” supplement, Annals of Plastic Surgery 90, no. 6S (2023): S667–s673, 10.1097/sap.0000000000003471. 37399487

[56] I. Ludolph , R. E. Horch , M. Harlander , et al., “Is There a Rationale for Autologous Breast Reconstruction in Older Patients? A Retrospective Single Center Analysis of Quality of Life, Complications and Comorbidities After DIEP or ms‐TRAM Flap Using the BREAST‐Q,” Breast Journal 21, no. 6 (2015): 588–595, 10.1111/tbj.12493. 26400037

[57] T.‐H. Shih and F. Xitao , “Comparing Response Rates From Web and Mail Surveys: A Meta‐Analysis,” Field Methods 20, no. 3 (2008): 249–271, 10.1177/1525822x08317085.

